# Recent Advances in Next Generation Snakebite Antivenoms

**DOI:** 10.3390/tropicalmed3020042

**Published:** 2018-04-15

**Authors:** Cecilie Knudsen, Andreas H. Laustsen

**Affiliations:** Department of Biotechnology and Biomedicine, Technical University of Denmark, Kongens Lyngby, DK-2800, Denmark; Cecilie.Knudsen@outlook.dk

**Keywords:** antivenom, snakebite, small molecule toxin inhibitors, oligonucleotides, antibodies, phage display, next generation antivenom, recombinant antivenom

## Abstract

With the inclusion of snakebite envenoming on the World Health Organization’s list of Neglected Tropical Diseases, an incentive has been established to promote research and development effort in novel snakebite antivenom therapies. Various technological approaches are being pursued by different research groups, including the use of small molecule inhibitors against enzymatic toxins as well as peptide- and oligonucleotide-based aptamers and antibody-based biotherapeutics against both enzymatic and non-enzymatic toxins. In this article, the most recent advances in these fields are presented, and the advantages, disadvantages, and feasibility of using different toxin-neutralizing molecules are reviewed. Particular focus within small molecules is directed towards the inhibitors varespladib, batimastat, and marimastat, while in the field of antibody-based therapies, novel recombinant polyclonal plantivenom technology is discussed.

## 1. Introduction

Snakebite is an epidemic of the rural tropics, which annually affects over 5 million people [1]. This leads to 1.84 million cases of envenoming and upper death toll estimates of 94,000, although experts generally agree that these numbers are likely underestimated [2]. Survivors are often afflicted by psychological disorders (e.g., post-traumatic stress) and left handicapped with amputations, blindness, or other sequelae [3,4,5,6,7]. The situation is further complicated by antivenom shortages and the undesirable traits of some antivenoms, including immunogenicity and low efficacy [8,9,10,11,12,13,14]. Antivenoms from hyperimmunized animals were first envisioned by A. Calmette and C. Phisalix in 1894 [15]. Since then, antivenoms have been optimized by including various purification steps, typically involving precipitation techniques, as part of their manufacture [16]. However, despite recent reports on innovative approaches for developing a new generation of antivenoms based on biotechnological methods, medicinal chemistry, and antibody technologies [17,18,19], plasma-derived antivenoms of animal origin remain the only effective treatment against snakebite envenoming [20,21]. Confronted with the severity of this neglected tropical disease through a campaign led by the Global Snakebite Initiative, Health Action International, Médecins Sans Frontières, the African Society of Venimology, and the Government of Costa Rica, the World Health Organization (WHO) reinstated snakebite envenoming on its list of Category A Neglected Tropical Diseases in 2017 [22,23,24,25] and set down a working group that will develop an official strategy for prevention and treatment of snakebite envenoming [26]. With the renewed international focus on snakebite envenoming, we here provide an updated overview of the most recent advances in the development of next-generation antivenoms that are not based on conventional animal immunization schemes. This review thus focuses solely on developments reported after 2016, as earlier important examples have been reviewed extensively elsewhere [17,18].

## 2. Small Molecule Inhibitors and Peptides

**Figure 1:**
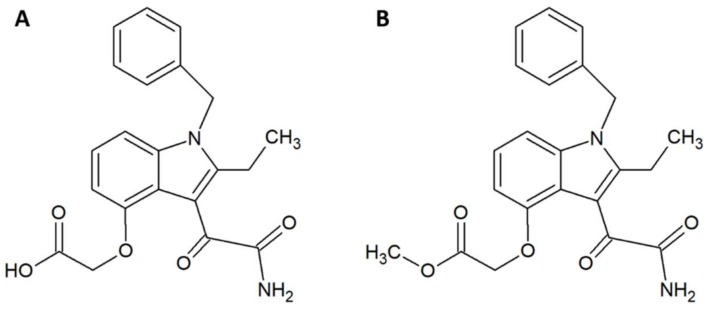
Chemical structures of (**A**) varespladib and (**B**) methyl-varespladib.

Within the field of medicinal chemistry, one interesting small molecule snake venom inhibitor to emerge recently is varespladib (Figure 1A) and the corresponding orally-available prodrug version, methyl-varespladib (Figure 1B). Varespladib previously went into clinical trials for a different indication, namely treatment of acute coronary syndrome [27], but never received approval by the US Food and Drug Administration [28,29]. A 2016 study by Lewin et al. demonstrated that nanomolar and picomolar concentrations of varespladib effectively inhibit the phospholipase A_2_ (PLA_2_) activities of selected snake venoms from various continents [28]. This activity against snake venom PLA_2_s is highly beneficial, as members of this toxin family are often poorly immunogenic [30] and hence may invoke only a poor immune response in production animals used for conventional antivenom manufacture. A poor immune response in production animals will in turn lead to a final antivenom product with limited efficacy against PLA_2_s. Mice pretreated with 4 mg/kg varespladib and subsequently envenomed with a lethal dose of *Micrurus fulvius* venom showed prolonged survival and reduced signs of haemorrhage [28]. This protection lasted for about 24 h, after which the effects wore off. When 4 mg/kg varespladib and a lethal dose of *Vipera berus* venom were co-injected subcutaneously, varespladib succeeded in increasing survival (3 of 7 mice survived, whereas all control mice died). A similar result was obtained when varespladib was injected with a slight delay after injection of *V. berus* venom. When 8 mg/kg varespladib was administered intravenously followed by subcutaneous administration of a lethal dose of *V. berus* venom, 100% of the treated mice survived [28]. In a final experiment, a group of rats challenged with *M. fulvius* venom by subcutaneous injection was rescued entirely when varespladib was administered intravenously within five minutes of the envenomation. Additionally, it was shown that varespladib suppressed the venom-induced rise in PLA_2_ activity and haemolysis of *M. fulvius* venom [28]. In a more recent study, varespladib was found to have a dose-dependent inhibitory effect on the PLA_2_ activities of *Deinagkistrodon acutus, Agkistrodon halys, Bungarus multicinctus*, and *Naja atra* venoms in vitro [29]. At 4 mg/kg, varespladib reduced the density of haemorrhagic plaques induced by *A. halys* and *D. acutus* venom, respectively, and decreased haemorrhage and oedema caused by all four venoms in vivo (oedema in mice treated with varespladib was decreased by 31–81% compared to control mice). Varespladib reduced the signs of venom-induced muscle damage, such as desmin degradation and serum creatine kinase levels. The ED_50_s for inhibition of lethality demonstrated that varespladib more effectively inhibited the viperid venoms of *D. acutus* (ED_50_ 1.14 µg/g) and *A. halys* (ED_50_ 0.45 µg/mg) compared to the elapid venoms of *B. multicinctus* (ED_50_ 15.23 µg/g) and *N. atra* (ED_50_ 22.09 µg/mg) [29]. It could be speculated that this is due to differences in PLA_2_ abundance or PLA_2_ subtypes between viperid and elapid venoms. As many snake venoms contain toxins (particularly from the PLA_2_ family) that exert their actions in synergy with other toxins and venom components [31], it could also be speculated that varespladib for certain snake venoms could interfere with important toxin synergisms leading to an inhibition of overall venom toxicity. However, not all snake venoms rely extensively on PLA_2_s. Thus, a natural limitation exists for the usefulness of the drug. As an example, venom from the *Dendroaspis* genus is almost entirely devoid of PLA_2_s [32,33,34], and it is unlikely that varespladib would be useful against bites inflicted by snakes of this genus. Nevertheless, while varespladib in itself may have interesting applications, its corresponding prodrug, methyl-varespladib, can be formulated for oral administration, making it a potential first line of defence. As such, alone or in combination with other drugs, methyl-varespladib might be able to buy snakebite victims the time needed to reach appropriate treatment facilities, where additional antivenom treatment can be provided. Such an application warrants further studies of absorption and bioavailability subsequent to oral administration.

**Figure 2:**
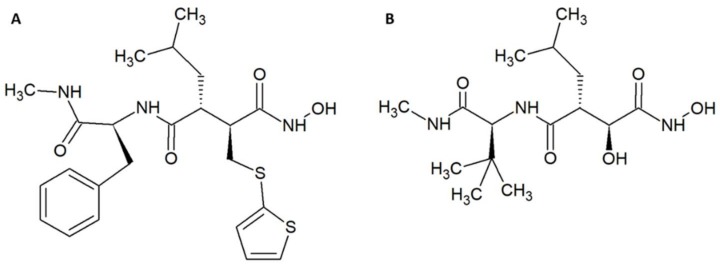
Chemical structures of (**A**) batimastat. (**B**) marimastat.

Other examples of promising small molecule inhibitors include the matrix metalloproteinase inhibitors batimastat (Figure 2A) and marimastat (Figure 2B) [35,36]. In a study by Arias et al., 200 µM of these molecules were incubated with 4 LD_50_s of *Echis ocellatus* venom and co-injected into the tail vein of CD-1 mice [37]. The molecules prolonged survival, but did not provide full protection. Nevertheless, administration of batimastat inhibited the haemorrhagic (IC_50_ = 30 µM), in vitro coagulant (IC_50_ = 0.05 µM), proteinase (IC_50_ = 2.6 µM), and defibrinogenating (IC_50_ = 200 µM) activities of the venom from an *E. ocellatus* specimen from Cameroon. IC_50_s for *E. ocellatus* venom from a specimen from Ghana were also determined; however, these vary somewhat from the values reported for the specimen from Cameroon. Fast administration of batimastat resulted in increased inhibition of haemorrhage. On the other hand, a delay in administration led to greater inhibition of defibrinogenation, which could be completely inhibited by a 60-min-delayed injection of 200 µL of 500 µM batimastat. Batimastat was more effective in inhibiting haemorrhagic activity than marimastat, and conversely marimastat was better at inhibiting defibrinogenating activity than batimastat. Five hundred micromoles of batimastat provided full protection against 1.5 LD_50_ of *E. ocellatus* venom when the venom was injected intramuscularly immediately followed by an intramuscular administration of batimastat. With a delay of 15–60 min in administration, batimastat no longer provided full protection, although it still prolonged survival [37]. Both compounds contain hydroxamate groups, which might by hydrolysed in plasma [38]. However, batimastat and marimastat have previously been investigated as potential cancer drugs. One study found that the half-life of batimastat was 19.1 days after intraperitoneal injection in humans (batimastat is not orally available) [39], and another study found that the half-life of marimastat after oral administration in humans is 8–10 h [40]. As these studies indicate that the half-life of batimastat and marimastat is on par with existing antivenoms, these small molecule inhibitors seem to be promising as supplements to existing antivenoms.

**Figure 3:**
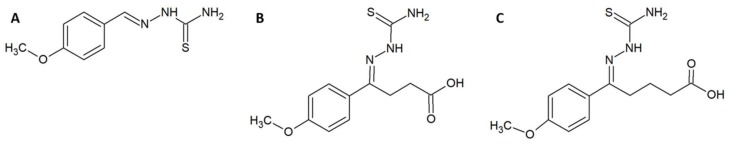
Chemical structures of (**A**) compound ‘2B’. (**B**) compound ‘5A’. (**C**) compound ‘5B’.

In a different study, Ferreira et al. utilised a combination of in vitro, in silico, and in vivo experiments in an attempt to design, synthesise, and evaluate enzyme inhibitors that could be used as fortifying supplements for antivenoms [41]. The rational design strategy for the small molecule enzyme inhibitors employed available sequence data on the metalloproteinase BpMP-I from *Bothrops pauloensis* and the crystal structure of the homologous BaP-I from *Bothrops asper* to create a three-dimensional (3D) model of BpMP-1, which was used to create a docking model for the inhibitors. Since these toxins are dependent on zinc ions, molecules containing zinc-chelating groups were generated and tested for their ability to inhibit the metalloproteinase in an azocasein assay [41]. Based on these results and the predicted docking geometries of these molecules and the toxin BpMP-I, two improved versions (5A and 5B) of the most promising molecule (2B) were designed (Figure 3). These were synthesised and once again evaluated in an azocasein assay. The two modified molecules were 38 and 1700 times more active than the original inhibitor, respectively, with IC_50_s of 78.12 µM and 1.77 µM. These improved compounds also decreased haemorrhagic activity in vivo. Incubation of 5B with whole venom from *B. pauloensis* 1:10 (*w*/*w*) (venom-to-inhibitor) followed by injection into mice completely reduced the haemorrhagic halo in the mice. If the venom was administered 10 min before the inhibitor, the haemorrhagic halo was, however, only reduced by 31%. Bioinformatic models predicted that the molecules possibly bind quite well to different snake venom metalloproteinases (SVMPs), although this was not explored experimentally. Finally, it was demonstrated that compound 5B interacts with Zn^2+^, which likely explains its inhibiting activity on Zn^2+^-dependent metalloproteinases [41].

Using a phage display approach, preliminary work on toxin-neutralizing peptides has also been reported [42]. Here, smaller peptides of varying lengths were first discovered using synthetic peptide phage display libraries, after which they were synthetized and tested for binding ability to their target toxin and related homologs. Peptide binders were discovered for a dendrotoxin from *Dendroaspis polylepis*, myotoxin II from *B. asper*, and α-cobratoxin from *Naja kaouthia*. For peptide 33535, a K_d_ was determined to 20 µM and truncated versions of this peptide (peptide 7 and peptide 8) were synthesised, which demonstrated comparable binding ability in a competitive ELISA setup [42]. No results on in vivo efficacy have, however, been reported.

Using one of the same peptide phage display libraries as Laustsen [42], Titus et al. employed phage display to find binders for a consensus PLA_2_ [43]. The PLA_2_ was based on a consensus sequence derived from alignments of available sequences of *Agkistrodon* PLA_2_s and mapped to an *Agkistrodon piscivorus piscivorus* PLA_2_ structure before being synthesised. Titus et al. selected binders capable of inhibiting *Agkistrodon piscivorus leucostoma* PLA_2_ activity in vitro as assessed by an EnzChek phospholipase A_2_ assay (Invitrogen). The four most promising binders inhibited 30–60% of the PLA_2_ activity. One of these binders was further tested against venom from *A. p. leucostoma* (again), *Crotalus adamanteus, Crotalus atrox, Crotalus scutulatus*, and *Agkistrodon contortrix laticinctus* using the same assay. Inhibition of PLA_2_ activities of these venoms was, however, only 30–40% [43], which leaves much room for improvement. Given that linear peptides typically have a poor half-life, much more work is needed before any analogues of the reported peptides may become as promising as varespladib.

**Figure 4:**
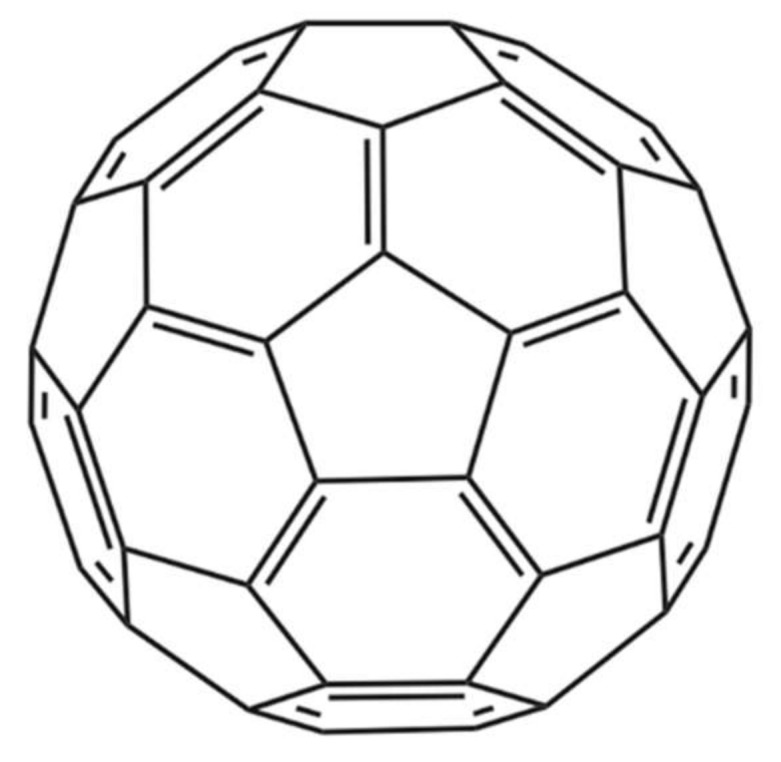
Chemical structure of the C60 fullerene [45].

Nanoparticles represent another technical avenue that could lead to the development of therapeutics that inhibit the activities of snake venom components. In one noteworthy study by Karain et al. [44], 4 µg/g of the nanoparticle C60 fullerene (Figure 4) prolonged survival of *Acheta domesticus* specimens (crickets) envenomed with *Crotalus oreganushelleri* venom. Twenty-four hours after injection, crickets that were administered the C60 fullerene, followed by administration of venom, had an average survival rate that was 15.7% higher than that of controls not receiving the C60 fullerene. After 48 h, the average survival rate was 25.0% higher [44]. These results warrant further studies in mammalian models. The C60 fullerene possesses several qualities desirable in an antivenom supplement, such as being cheap, stable, and having a great volume of distribution. However, the fact that it cannot be given intravenously due to its hydrophobicity and must be given orally or injected intraperitoneally dissolved in olive oil or a similar vehicle decreases its immediate usefulness for treatment of envenoming and affects its pharmacokinetics. While C60 becomes detectable almost instantly in the blood, it still takes 8 h for it to reach maximal concentration when administered orally in rats [44]. This could prove problematic in the case of fast-acting venoms and makes C60 less readily applicable for snakebite treatment.

**Figure 5:**
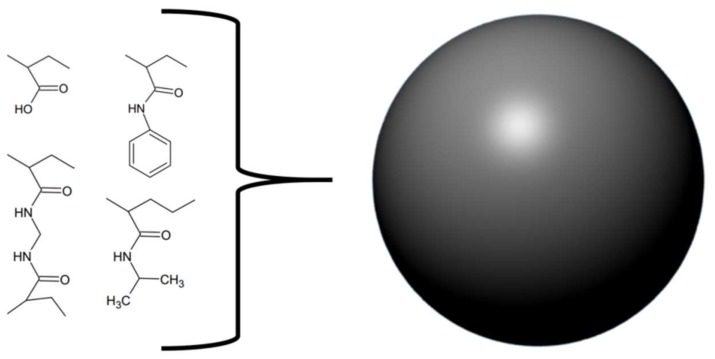
Schematic overview of the molecular components used by O’Brien et al. [46] to assemble the nanoparticles.

Similar to Ferreira et al. [41], O’brien and colleagues [46] chose to follow an approach of rationally designing, synthesising, and evaluating a molecule capable of neutralising venom activities. They synthesised various nanoparticles from different mixtures of four components and tested their abilities to neutralize the PLA_2_ activity of whole venoms from *Bungarus caerulus, Naja mossambica*, and *Apis mellifera* (Figure 5). The goal was to find a nanoparticle capable of broadly neutralising the effects of PLA_2_s from various snake venoms. Using this approach, the authors developed a non-cytotoxic nanoparticle with long dissociation rates for PLA_2_s. This nanoparticle is devoid of phospholipids and does therefore not act as a substrate for PLA_2_s. Instead, results indicate that the nanoparticle exerts its function by interacting directly with PLA_2_s rather than with lysophosphatidylcholine (a product of PLA_2_ activity responsible for inducing haemolysis) [46]. It should, however, be noted that such nanoparticles have never been tested in vivo, and it is unknown whether their in vitro efficacy will translate well into a preclinical setting.

A trend within the studies showcased here is the focus on enzyme inhibitors. Seeing as many toxins belong to the same enzymatic protein families (e.g., snake venom metalloproteinases, serine proteinases, and PLA_2_s), and since these toxic enzymes share very similar substrates within these families, the strategy of targeting enzymatic activity with a substrate mimetic seems logical. Most enzymes have catalytic clefts, which are feasible targets for engineered and naturally occurring inhibitors. Hence, it is unsurprising that much prior work was focused exactly on inhibition of enzymatic toxins [17].

## 3. Oligonucleotides and Antibodies

Although the main work was performed on a cone snail toxin, and not a snake toxin, another approach to developing novel compounds for snakebite envenoming therapy focuses on the use of oligonucleotide-based aptamers, which have found various applications, including in therapeutics [47]. In 2017, El-Aziz and colleagues published an article arguing for favourable characteristics of oligonucleotides, including low immunogenicity, small size, thermal stability, biocompatibility, and standardised production methods [48]. Oligonucleotides are devoid of many of the drawbacks associated with antibody production by immunisation (e.g., use of production animals, long production time, poor immunogenicity of many smaller toxins, and cost of production) and with the treatment itself (e.g., immunogenicity of animal-derived antibodies, limited shelf-life, need for refrigeration, and potential lack of specificity). On this basis, El-Aziz et al. sought to find an oligonucleotide capable of neutralising the activity of the αC-conotoxin PrXA from the cone snail species, *Conus parius*. One of the tested oligonucleotides proved capable of inhibiting the activity of αC-conotoxin PrXA in vitro, but at the tested doses (0–0.25 µg oligonucleotide/g mouse bodyweight) could only provide prolonged survival in vivo and not full protection. When administered at higher concentrations, the oligonucleotide did, however, provide full protection in vivo. The ED_50_ against lethality for the oligonucleotide was determined to be 0.18 µg/g mouse bodyweight when administered intraperitoneally, and 0.22 µg/g mouse bodyweight when administered subcutaneously. As the oligonucleotide was unable to inhibit a different blocker (waglerin) of the muscle nicotinic acetylcholine receptor targeted by the conotoxin, the oligonucleotide was assumed to be specific for αC-conotoxin PrXa [48]. Another benefit of working with oligonucleotides in the lab setting includes the low cost of small-scale synthesis for research and development (R&D) purposes, which makes it easy for researchers to quickly evaluate a large range of molecules at limited cost. However, before evaluating the use of oligonucleotides in the clinical setting, more studies evaluating their cost of manufacture in larger scale are needed.

**Figure 6:**
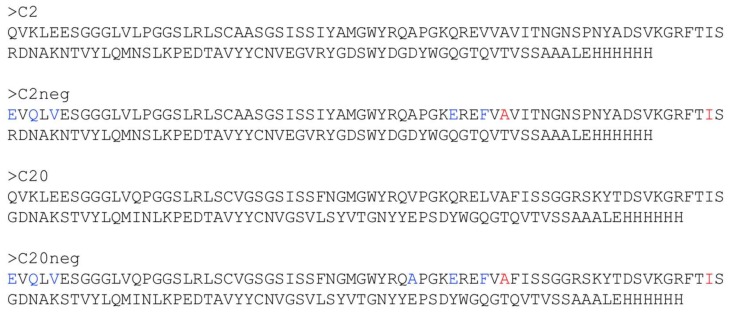
Overview of mutations introduced in V_H_Hs by Anderson et al. to improve the thermal stability of the C2 and C20 nanobodies [51]. The sequences of the wild-type antibodies C2 and C20 are shown. The sequences of the mutated C2neg and C20neg antibodies are also shown, with mutations highlighted in blue. Finally, C2neg and C20neg were further mutated by the introduction of additional disulphide bridges to form C2neg+ and C20neg+. This was accomplished by mutating the amino acids highlighted in red into cysteines. Adapted with the author’s permission.

In the field of recombinant antivenoms, attention to the use of camelid V_H_Hs (also known as nanobodies) as therapeutic agents has increased. This is likely due to their stability (thermal, chemical, pH), solubility, high target specificity and affinity, and good expression levels in prokaryotic expression systems, which make them attractive as therapeutic molecules for the treatment of envenomation [49,50]. Due to their small size, V_H_Hs have a relatively large volume of distribution, but they have been shown to have relatively short half-lives in vivo. Recent advances within the development of recombinant antivenoms includes a study by Anderson et al., in which the authors set out to improve the stability of two V_H_Hs by making them more heat-resistant [51]. The suboptimal thermal stability of existing antivenoms is currently compensated for by using cold-chain transportation and storage. The necessity of a cold chain is both costly and may be outright unavailable in certain regions. Lyophilisation of antivenom is a commonly used alternative to cold chains but can potentially lead to protein denaturation and prolonged time to treatment as the antivenom must be reconstituted before use. Improved thermal stability might negate this need for a cold chain and lyophilisation of antivenoms [51]. The starting points of Anderson’s study were the V_H_Hs C2 and C20, which had a previously-demonstrated ability to neutralize the toxic effects caused by α-cobratoxin from *N. kaouthia* venom [51,52]. The authors introduced mutations known to enhance the thermal stability of V_H_Hs and further introduced an additional disulphide bridge in each antibody (also known generally to lead to increased stability) (Figure 6). With one exception, all mutated binders retained the affinities for α-cobratoxin of the original binders. Additionally, refolding and retention of activity after thermal stress was improved for all mutants. For the C20 mutants, aggregation due to thermal stress was also decreased. Finally, the melting temperatures were improved from 71 °C for C2 to 86 °C for the best C2 mutant, and from 60 °C for C20 to 75 °C for the best C20 mutant [51].

**Figure 7:**
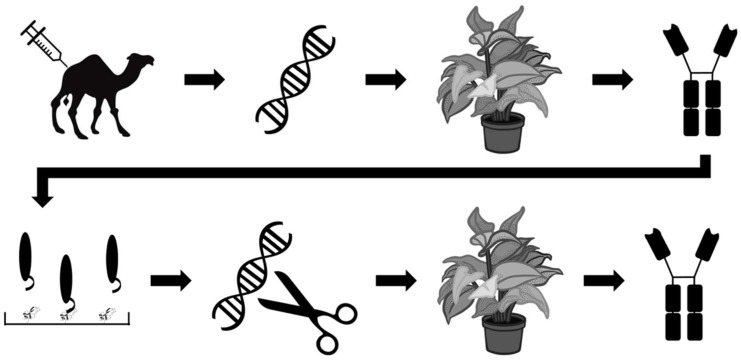
Schematic overview of the strategy employed by Julve Parreño et al. [53]. Dromedaries were immunised with a mixture of venoms. V_H_H sequences were extracted from the immunised animals, cloned into a viral vector, and used to infect *Nicotiana benthamiana* specimens. The plant-expressed V_H_Hs were evaluated in vivo and subjected to phage display experiments involving four venom fractions from *Bothrops asper* (representing the four important major toxin families of this venom) to accumulate high affinity V_H_Hs. The 36 best V_H_H binders were converted to human-dromedary chimeric antibodies, which were expressed in *N. benthamiana* as well and tested in vivo.

Other efforts within the field of recombinant antivenom are aimed at optimizing the production rather than the stability of antivenoms. One such effort is reported by Julve Parreño et al., who explored the possibility of producing so-called ‘plantivenoms’ [53]. The researchers used in planta production of polyclonal antibodies derived from V_H_H sequences of dromedaries immunised with mixtures of venoms from *Crotalus simus, C. scutulatus,* and *B. asper*. This was accomplished by the insertion of dromedary V_H_H sequences into a genetically-modified vector derived from tobacco mosaic virus, which was subsequently used to infect *Nicotiana benthamiana* specimens via *Agrobacterium tumefaciens* bacteria and stimulate antibody production. The authors demonstrated that the results were reproducible and that the plantibodies proved to be capable of binding venom components from *C. simus, C. scutulatus,* and *B. asper* venoms, but not venom components of *Naja nubiae* and *N. mossambica* venom. To further improve their plantibodies, the researchers employed phage display technology to select the best *B. asper* V_H_H binders against four different venom fractions, representing the four most important toxin families in this venom. The 36 most promising V_H_H binders were selected and converted into chimeric antibodies composed of the human IgG constant regions of the heavy chains and the V_H_Hs (Figure 7). This optimized oligoclonal plantivenom showed a similar binding pattern to the existing *B. asper* antivenom and could neutralise the haemorrhagic, PLA_2_, proteinase, and lethal activities of *B. asper* venom, but not the in vitro coagulant activity. However, the equine-derived *B. asper* antivenom used as a control had a superior efficacy, as demonstrated by their respective ED50s (neutralisation of lethality: 3.1 mg antivenom/mg venom versus 43.2 mg plantivenom/mg venom) [53]. Under the given experimental conditions, the plantivenom successfully prevented lethality in all mice at a dose of 61.24 mg plantivenom/mg venom. While improvements are needed to increase the plantivenom’s titer and neutralizing capacity of the coagulant activity of *B. asper* venom to become on par with existing antivenoms, this study clearly demonstrates promising results for a highly innovative approach to antivenom development.

Julve Parreño et al. claim that in planta production may serve as a cost-effective alternative to conventional antivenom manufacture and even to the manufacturing of recombinant monoclonal antibodies [53]. However, the researchers do not provide comprehensive argumentation for their cost evaluations, and such viewpoints therefore deserve further scrutiny. The drawbacks of plants as expression systems for animal/human antibodies particularly include low protein yields, non-human glycosylation patterns, and the need for isolating and purifying the antibodies from the rigid plant matrix, which is high in cellulose, lignin, and other polymeric macromolecules. The authors achieved a yield of 0.2 g antibodies per kg of plant leaves [53], which is indeed impressive, as limited efforts had been applied to optimising expression. However, this does not compare favourably with standardised mammalian cell cultivation approaches, which routinely achieve yields of 5 g/L of correctly folded and secreted IgGs [20], with exceptional examples even reaching 27 g/L [54]. Julve Parreño et al. also argue that the production of oligoclonal antibody mixtures by parallel batch expression is not economically feasible without compromising efficacy, as many antibodies are needed to target the large arsenal of toxins present in snake venoms [53]. In itself, this statement may not be incorrect. However, with the recent introduction of oligoclonal expression systems for chinese hamster ovary (CHO) cells [55,56,57], cost simulations for the manufacture of oligoclonal antibody mixtures recombinantly expressed by mammalian cells in a single-batch setup demonstrate that such mixtures can be produced cost-competitively compared to current antivenom manufacture [58,59]. In planta expression of antibody mixtures may therefore very well serve an important purpose within development. Yet it remains to be seen whether this approach is in fact economically attractive. A flag should at least be raised, given that no antibody-based therapy relying on in planta expression has ever entered the market [60] and previous attempts to bring the Ebola therapeutic, ZMapp (consisting of three human IgGs originally expressed in *N. benthamiana*) [61,62], to the clinic encountered scale up and manufacturing challenges with the plant-based expression system.

The field of human antibody fragments has for many years been championed by groups from Brazil and Mexico [63,64,65,66,67,68,69,70]. In this field, Silva et al. reported the use of phage display technology to select human scFvs against components from the venoms of *Crotalus durissus terrificus* and *Bothrops jararacussu* [71]. Based on an ELISA screening approach, three of these scFvs (B7, C11, and E9) were selected due to their cross-reactivity to components in both venoms. The researchers demonstrated that the scFvs were capable of neutralising the haemolytic and plasma-clotting activities of crotalic and bothropic venoms to different extents, and that the scFvs had the greatest effect on venom activity when they were used in combination. Haemolytic activity was fully inhibited by the combination of scFvs, while plasma-clotting was decreased but not fully prevented. Furthermore, the scFvs prolonged the survival of envenomed mice at ratios of 1:1 (*w*/*w*), but were maximally able to provide protection against lethality for 25% of the mice [71]. More research is thus needed to discover and engineer such human scFvs to gain improved efficacy.

## 4. Conclusions and Perspectives

Renewed interest in snakebite envenoming from international organisations, such as the WHO, could contribute to spreading awareness of this neglected tropical disease and facilitate increased research efforts and the development of new treatments. Current trends in such efforts include the investigation of the utility of several small molecule inhibitors, which are currently being evaluated for their ability to neutralise the effects of enzymatic toxins. Perhaps the most promising of these small molecule inhibitors, varespladib, may have the potential to become a broad-spectrum orally administered first line of treatment for snakebite victims, or possibly an anti-PLA_2_-specific supplement to conventional antivenom therapy. While small molecule inhibitors might be particularly well-suited for targeting enzymatic toxins, such as PLA_2_s and proteinases, smaller non-enzymatic toxins (such as three-finger toxins) may better be targeted by antibody-based or antibody-like therapeutics. It is therefore likely that increased research efforts on nanobodies and human antibody formats will occur within the next few years. This not only represents an opportunity for innovation within snakebite antivenoms, but it may also facilitate more research efforts on the development of therapies against envenomings by other animals, such as scorpions and spiders.
